# Synthesis and Biological Evaluation of HDAC Inhibitors With a Novel Zinc Binding Group

**DOI:** 10.3389/fchem.2020.00256

**Published:** 2020-04-15

**Authors:** Junquan He, Songsong Wang, Xingang Liu, Ruili Lin, Fang Deng, Zhong Jia, Chenghong Zhang, Zhao Li, Hongtian Zhu, Lei Tang, Pingrong Yang, Dian He, Qingzhong Jia, Yang Zhang

**Affiliations:** ^1^Materia Medica Development Group, Institute of Medicinal Chemistry, Lanzhou University School of Pharmacy, Lanzhou, China; ^2^NMPA Key Laboratory for Quality Control of Traditional Chinese Medicine, Gansu Institute for Drug Control, Lanzhou, China; ^3^The Second Hospital of Hebei Medical University, Shijiazhuang, China; ^4^College of Pharmacy, Hebei Medical University, Shijiazhuang, China; ^5^Pharmacy Department, Lanzhou Second People's Hospital, Lanzhou, China; ^6^School of Pharmaceutical Sciences, Chongqing University, Chongqing, China

**Keywords:** HDAC inhibitor, Novel ZBGs, Anti-tumor, Molecular docking, MD simulation

## Abstract

Vorinostat (SAHA) with great therapeutic potential has been approved by the FDA for the treatment of cutaneous T-cell lymphoma as the first HDACs inhibitor, but the drawbacks associated with hydroxamic acid group (poor stability, easy metabolism, weak binding ability to class IIa isozymes, and poor selectivity) have been exposed during the continuous clinical application. Based on the pharmacophore of HDAC inhibitors, two series of compounds with novel zinc binding group (ZBG) were designed and synthesized, and the antitumor bioactivities were evaluated in four human cancer cell lines (A549, Hela, HepG2, and MCF-7). Among the synthesized compounds, compounds **a6**, **a9**, **a10**, **b8**, and **b9** exhibited promising inhibitory activities against the selected tumor cell lines, especially compounds **a9** and **b8** on Hela's cytostatic activity (**a9**: IC_50_ = 11.15 ± 3.24 μM; **b8**: IC_50_ = 13.68 ± 1.31 μM). The enzyme inhibition assay against Hela extracts and HDAC1&6 subtypes showed that compound **a9** had a certain broad-spectrum inhibitory activity, while compound **b8** had selective inhibitory activity against HDAC6, which was consistent with Western blot results. In addition, the inhibitory mechanism of compounds **a9** and **b8** in HDAC1&6 were both compared through computational approaches, and the binding interactions between the compounds and the enzymes target were analyzed from the perspective of energy profile and conformation. In summary, the compounds with novel ZBG exhibited certain antitumor activities, providing valuable hints for the discovery of novel HDAC inhibitors.

## Introduction

Cancers with increasing incidence and mortality have been the leading cause of death, seriously threating the public health and making the design and discovery of chemotherapeutic agents particularly important (Evans et al., 2017). Although substantial progress has been made in the antitumor drugs, drug resistance and high toxicity limit their clinical application (Hughes and Andersson, 2015; Yan and Li, 2018; Santiago-O'farrill et al., 2019). In addition, various factors are involved the development of the tumorigenesis, especially the changes in gene expression associated with mutation, loss, and rearrangement, making the mechanism much more complicated (Wenbo and Wang, 2017). Moreover, with few exceptions, it is difficult to target therapeutically these gene changes (Kanwal et al., 2015; Chen et al., 2019). With the continuous development of epigenetic mechanisms, the increasing evidence suggests that the epigenetic dysregulation of gene expression interacts extensively with the tumor initiation and progression (Connolly and Stearns, 2012; New et al., 2012; Seidel et al., 2012; Abdelfatah et al., 2016), which can be comprehensively investigated as a novel anticancer drug development strategy. Epigenetic modifications refer to the heritable alterations in gene expression unrelated to the DNA sequences, and the histone acetylation catalyzed by histone acetyltransferases (HATs) and histone deacetylases (HDACs) has become a major component of epigenetic research field (Glozak and Seto, 2007; Kouzarides, 2007; Zhang et al., 2017). Significantly, unlike the modification in DNA sequence, the epigenetic changers are reversible, showing great potential in targeting epigenetic regulation of gene for the discovery of new anticancer agents.

HDAC family consists of 18 subtypes, which are further classified into four groups according to protein size, cellular localization, and the homology to yeast HDAC proteins: Classes I (HDAC1-3, and HDAC8), Classes IIa (HDAC4, 5, 7, and 9), Class IIb (HDAC6 and 10), and Classes IV (HDAC11) are metal dependent, while class III (Sirtuins) are NAD^+^ dependent (Gregoretti et al., 2004; Balasubramanian et al., 2009). The HDAC proteins are closely related to the basic cellular processes, functions, and the disease states, especially cancers, but the biological functions of individual isoforms in cell and cancer biology are still not well-elaborated. The metal-dependent HDACs could regulate the expression and bioactivities of cancer-related proteins involved in transcription, tumor suppression, and cell signaling, and can catalyze a variety of substrates including nucleosomal histones and non-histone, influencing the interactions between protein-protein and protein-DNA as well as the transcriptional process, which make such metalloproteins gradually become important targets for cancer treatment (Bolden et al., 2006; Glozak and Seto, 2007; Kouzarides, 2007; Suresh et al., 2017).

Currently, a large number of HDAC inhibitors with diverse molecular skeletons have been reported, which conform the common pharmacophore features consisting of the cap group (Cap), connect unit (CU), the linker moiety (Linker), and a zinc binding group (ZBG) (Miller et al., 2003; Bolden et al., 2006; Dokmanovic et al., 2007; Thaler and Mercurio, 2014; Yoon and Eom, 2016). In fact, several HDAC inhibitors have been applied for treatment of T-cell lymphoma, such as SAHA (suberoylanilide hydroxamic acid, Vorinostat) (Negmeldin and Pflum, 2017) and Belinostat (Poole, 2014), and Panobinostat (Garnock-Jones, 2015). Unfortunately, these FDA approved drugs are relatively non-selective and inhibit most of the zinc-dependent HDAC subtypes (Yang, 2011; Qiao et al., 2013), which could cause several mild or severe toxic effects associated with the treatment, such as dehydration, thrombocytopenia, anorexia, and cardiac arrhythmia (Chakrabarti et al., 2016; Buonvicino et al., 2018; Cosenza and Pozzi, 2018; Laino et al., 2019). In addition, due to the physicochemical properties of hydroxamic acid group as the ZBG, some drawbacks have been exposed, including poor stability, easy metabolism, weak binding ability to class IIa isozymes, and poor selectivity, making the design and discovery of HDAC inhibitors with novel zinc binding group (ZBG) more necessary (Di Micco et al., 2008; Botta et al., 2011; Burli et al., 2013; Zhao et al., 2018; Banerjee et al., 2019).

In this study, based on the molecular scaffold of SAHA, two series of compounds were designed and synthesized with ethanolamine and 1-amino-2-propanol as the ZBG, which were subject to MTT assay, enzyme inhibition experiment, and Western blot experiment to evaluate the biological activities. Furthermore, molecular docking and molecular dynamic (MD) simulation were applied to study the difference in inhibitory mechanism of compounds **a9** and **b8** in HDAC1 and HDAC6 at the atomic level. Finally, we found that the binding pattern of compound **a9** in HDAC1's active pocket was similar to that of SAHA, and due to the complementary energy contribution of the residues located in the binding sites of HDAC1&6, the binding free energies of compound **a9** on the two targets were similar, which might be the reason for its non-selective inhibitory activities against HDAC1&6. However, compared with compound **a9**, the binding pattern of compound **b8** on the targets were different, and contributed to the differences in energy contributions of amino acids at HDAC1&6, resulting in the selective inhibition against HDAC6 isoform.

## Materials and Methods

### Materials and Instruments

All reagents and solvents should meet the standards of analytical reagent before use, and the melting points of all the synthesized compounds were determined in open capillaries using Shengyan electrothermal PIF YRT-3 apparatus without correction. Bruker AM-400 was applied to record ^1^H NMR and ^13^C NMR spectra, and LCQ Deca XP plus was used to determine the ESI/MS spectra. In this study, human cancer cell lines A549, MCF-7, HepG2, Hela and the normal cell WI-38 were purchased from Cell Resources Center of Shanghai Institutes for Biological Science (Chinese Academy of Sciences), which were cultured on the basis of supplier's instructions. DMEM (Dulbecco's modified Eagle medium), FBS (fetal bovine serum) were obtained from Hyclone (Shanghai, China), and MTT [3-(4, 5-dimethylthiazol-2-yl)-2, 5-diphenyltetrazolium bromide] were provided by Sigma (Beijing, China).

All reagents and solvents are reagent level or are purified by standard methods prior to use. HDAC Inhibitor Drug Screening Kit was purchased from BioVision, and acetyl-histone H3, acetyl-α-tubulin and β-actin were obtained from AFFINITY BIOSCIENCE. Fluor de Lys^®^ HDAC1 Assay kit (BML-AK511, Enzo^®^ Life Sciences) and Fluor de Lys^®^ HDAC6 Assay kit (BML-AK516, Enzo^®^ Life Sciences) were applied to determine the inhibitory activities of the compounds against HDAC1 and HDAC6 subtypes.

### Chemistry

**Figure 1:**
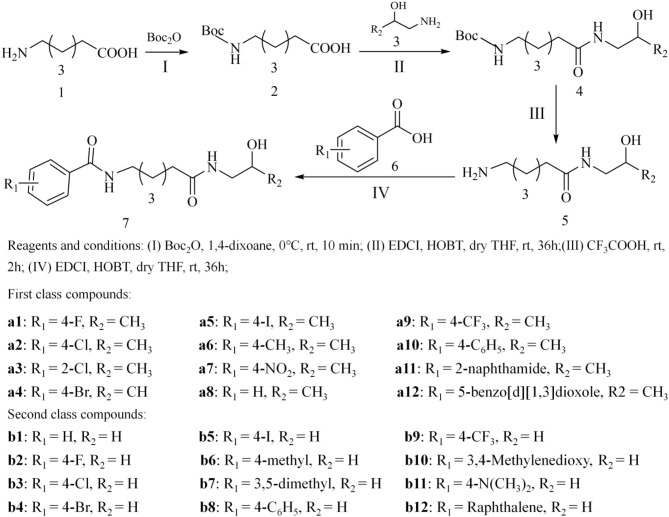
Synthetic route of the target compounds.

The tittle compounds **a1-a12, b1-b12** were synthesized based on the reported synthetic method, mainly including four experimental steps shown in Figure 1 (Zhang et al., 2015). (I) the amino group in hexaaminocaproic acid (compound **1**) was protected by (Boc)_2_O reagent, and the details were as follows: compound **1** was dissolved in 5 mL water containing 6 mmol NaOH; then 10 mL 1, 4-dioxane was added to the mixture, and (Boc)_2_O reagent (6.6 mmol) was added during the stirring process under ice bath condition; after 10 min, the reaction was transferred to the room temperature environment, and then stirred for 10 h to obtain compound **2** (tan oil). (II) compound **4** was obtained by condensation reaction of compound **2** and compound **3** with 1-(3-dimethylaminopropyl)-3-ethylcarbodiimide (EDCI) and 1-hydroxybenzotriazole (HOBT) as the coupling agents, and experimental details were as follows: compound **2** was dissolved in DMF together with EDCI and HOBT, and stirred for 1 h; then compound **3** was added to the mixed reaction solution, and the reaction was stirred at room temperature for 36 h; all the progress of the reaction was monitored by thin layer chromatography until the end of the reaction. (III) compound **4** was dissolved in trifluoroacetic acid and stirred to remove the Boc group, and the specific experimental operation was as follows: compound **4** and 2 mL trifluoroacetic acid were added to a round bottom flask, stirred for 2 h, and the progress of the reaction was monitored by thin layer chromatography; after the completion of the reaction, the excess trifluoroacetic acid was removed by reduced pressure distillation. (IV) the tittle compounds were obtained by further condensation reaction of substituted benzoic acid and compound **5**, and the specific experimental operation was consistent with step II.

Separation and purification of the compounds were performed on flash column chromatography with silica gel (200–400 mesh), and analytical thin-layer chromatography (TLC) was conducted on Fluka TLC plates (silica gel 60 F254). Additionally, all the synthesized compounds were characterized by ^1^H NMR, ^13^C NMR, and ESI-MS.

### Assessment of Cytotoxicity

The antitumor bioactivities of the tittle compounds *in vitro* were firstly evaluated against four different human tumor cell lines [breast lung cancer (A549), cervical cancer (Hela), liver cancer (HepG2), breast cancer (MCF-7)] via MTT assay, and a normal cell line [human lung fibroblast (WI-38)] was applied to assess the safety of the synthesized compounds. Briefly, the selected cell lines were cultivated in RPMI1640 medium supplemented with 10% fetal bovine serum under the environment of 37°C, 5% CO_2_, and 90% humidity, and the antibiotics (penicillin/streptomycin) and antifungals were added to prevent cell contamination during the culture process. In this study, the tested compounds were diluted to the required concentration with culture medium, and growth inhibitory effects against the cell lines of the tittle compounds were determined by MTT colorimetric assay. Afterwards, the cells (100 μL, 1 × 10^5^ cells mL^−1^) were seeded on 96-well plates and kept to adhere for 12 h, and then the medium was replaced with fresh media containing the synthesized compounds with different concentrations (12.5, 25, 50, 100, and 200 μmol L^−1^), which were transferred to the incubator and cultured for another 48 h. Then, MTT phosphate buffer solution (PBS) (10 μL, 5 mg·mL^−1^) was added to the 96-well plates, and the medium was replaced with DMSO (150 μL). The microplate reader was adopted to record the absorbance at 490 nm for each well of the plates. In this MTT assay, SAHA was used as the reference drug.

### Apoptosis and Cycle Arrest of Hela Cells Induced by Compounds a9 and b8

Hela cells were cultured in RPMI 1640 medium supplemented with 10% fetal bovine serum under environment of 37°C, 5% CO_2_, 90% humidity, which were then transferred to the 6-well plate and cultured for 48 h. The medium was removed from the wells and the Hela cells were processed with compound **a9** and **b8** with different concentrations. Afterwards, Hela cells were detached using 0.25% trypsin–EDTA (0.5 mL) and then re-suspended in medium (4 mL) before centrifugation (1000 rpm for 5 min). Cell pellets were washed twice by PBS (2 mL) to remove the residual medium, and the cells were fixed in cold 70% ethanol. To assess the apoptosis, the double Annexin V-FITC/PI (Solarbio) immunofluorescence labeling method was applied, and Beckman Coulter flow cytometer was used to monitor the fluorescence intensity. Afterwards, the collected Hela cells were stained with propidium iodide (PI) in the dark for 30 min at 37°C, and the DNA content of Hela cells was analyzed using BD FACS verse™ flow cytometry.

### Enzyme Inhibition Assay

Hela nuclear extracts (HDAC Inhibitor Drug Screening Kit, BioVision) were adopted to evaluate the enzyme inhibitory activities of compound **a9** and **b8** with SAHA as the reference, and the details were as follows: (1) compounds **a9** and **b8** were dissolved in DMSO and diluted to the desired concentrations with double distilled water (ddH_2_O); (2) according to the instruction of kit, 10 × HDAC Assay Buffer (10 μL), Hela Nuclear Extract (2 μL), HDAC Substrate (5 μL), and ddH_2_O (33 μL) were proportionally prepared into the reaction mixture, and 50 μL reaction mixture was added to the 96-well plate, which was transferred to CO_2_ incubator and cultured for 30 min at 37°C; (3) after that, 10 μL lysine developer was added to the 96-well plate, and mixed well, which were incubated for additional 30 min; (4) microplate reader was selected to determine the fluorescence intensity at excitation wavelength of 360 nm and emission wavelength of 450 nm.

Furthermore, the inhibitory bioactivities of compounds **a9** and **b8** against HDAC1 and HDAC6 subtypes were also evaluated using the commercially available HDAC assay kits, and in this experiment, Fluor de Lys^®^ HDAC1 Assay kit (BML-AK511, Enzo^®^ Life Sciences) and Fluor de Lys^®^ HDAC6 Assay kit (BML-AK516, Enzo^®^ Life Sciences) were selected. All the assay components were diluted in TrisHCl buffer (50 mM TrisHCl, pH 8.0, 137 mM NaCl, 2.7 mM KCl, 1 mM MgCl_2_), and SAHA was used as the control compound. The generated fluorescence after the deacetylation of HDAC could be detected at the wavelengths of 485 nm (excitation) and 530 nm (emission) using the microplate reader (PerkinElmer, USA). The dose response of inhibition experiment was carried out in triplicate, and the IC_50_ data was calculated by GraphPad Prism 5.

### Western Blot Analysis

Hela cells were seeded in 6-well plates (5 × 10^5^/well) overnight to allow the cells to adhere, and then the medium solution was removed, which were then further incubated with compounds **a9** and **b8** at the concentrations of 0, 20, and 40 μM for 48 h. Afterwards, the Hela cells were collected using centrifugation, and were then lysed by the lysate, which were carried out further centrifugation at 12,000 rpm for 30 min. The protein of supernatant obtained by centrifugation was quantified using the BCA kit (Beijing Solarbio Science & Technology Co., Ltd.), and the protein extracts were mixed with the SDS-PAGE protein loading buffer (Beijing Solarbio Science & Technology Co., Ltd.), which were subject to 100°C water bath for 10 min to sufficiently denaturate. The gel was gelatinized using the SDS Gel Kit (Beijing Solarbio Science & Technology Co., Ltd.) and protein extracts were separated by protein SDS-polyacrylamide gel electrophoresis and then transferred to Immobilon-NC Membrane (Beijing Solarbio Science & Technology Co., Ltd.). After the blocking with 5% non-fat dried milk in TBS for 90 min at room temperature, the membranes were incubated overnight with specific primary antibodies (AFFINITY BIOSCIENCE) at 4°C, which were further incubated with secondary antibodies for 2 h after washing three times with TBST. The chemiluminescence analysis system was applied to label the target protein with a specific primary antibody and the target protein was detected using a specific antibody.

### Molecular Docking

#### Protein Preparation

Crystallographic structures of HDAC1&6 could be available in protein data bank [PDB entry: 5ICN (Watson et al., 2016), 5EDU (Hai and Christianson, 2016)], and *Protein Preparation Wizard* module in Maestro v. 9.0 (2009) was applied to process the whole protein crystals before generating the docking grid, including adding hydrogen atoms, assigning protonation states and partial charges with OPLS-2005 force field. Afterwards, the *Receptor Grid Generation* tool was adopted to define the docking grid using the TSA in 5EDU and the novel peptide inhibitor in 5ICN as the references.

#### Ligands Preparation

The structures of the ligands were drawn by ChemBioDraw and saved in ^*^.sdf format, which were then preprocessed by *LigPrep* with OPLS3 force fields to create the 3D structures and minimize the energy of the ligand conformations. Then, Epik v. 2.0 (2009) was used to generate and assign the ionized states of the ligands.

#### Molecular Docking

Standard precision (*SP*) docking in Glide v.5.5 (2005) was applied to determine the docking poses of all the studied systems with default parameters, and 5,000 poses were generated during the initial phase of the docking calculation, out of which the best 400 poses were selected for energy minimization. Then, the initial docked poses of the constructed complexes were selected on the basis of spatial coordinates of TSA and peptide inhibitor in the protein crystals as well as the docking scores provided by Glide v.5.5 (2005).

### Molecular Dynamic (MD) Simulation

#### Systems Preparation

Prior to MD production, the components of the studied complexes (receptor, ligands, Na^+^, Cl^−^, and the zinc ion) were processed by force field parameters. First, the geometry optimization and electrostatic potential calculation of the ligands were performed using Gaussian 09 at HF/6-31G^*^ level, and the generated frcmod and mol2 format of the ligands were used to assign *gaff* atom types and the *RESP* partial charges via *antechamber* model in AMBERTOOLS16 (AMBER v. 16, 2016). Then, the coordinate files (.inpcrd) and topology file (.prmtop) were generated using the *LEaP* module in AMBERTOOLS16 with corresponding force field (*ff14SB*^53^ for the protein, general amber *force fields* for the ligands, 126-4 model for the zinc ion, and parameters from *Joung*'s work for Na^+^ and Cl^−^) to generate the corresponding coordinate files (.inpcrd) and topology file (.prmtop) through *LEaP* module in AMBER v. 16 (2016).

#### MD Production

Before the MD simulation, the constructed research systems were subjected to the initial energy minimization via two steps: (**1**) solute atoms were processed by harmonic restraint (force constant = 10 kcal·mol^−1^·Å^−2^); (**2**) all the atoms were released to move freely. In the two steps, energy minimization was conducted using the steepest descent method for the first 5,000 steps and the conjugated gradient method was applied for the following 5000 steps. Then, each studied system was heated from 0 to 100K and then gradually to 310K with the protein restrained over 100ps in the NVT ensembles. Afterwards, 5 ns unrestrained equilibration at 310 K was performed to equilibrate the periodic boundary condition of the studied complexes. Finally, the unrestrained 150 ns production simulation was carried out for the constructed systems in NPT ensembles at the temperature of 310 K and the pressure of 1atm.

#### Binding Free Energy and Per-Residue Binding Energy Decomposition Calculation

Molecular mechanics/generalized born surface area (MM/GBSA) method was adopted to calculate the binding free energy (ΔG_MM/GBSA_) using single molecular dynamic trajectory regardless of entropic influence between the docked ligands and HDACs. Herein, 500 snapshots were extracted from the equilibrium trajectories (100–150 ns) for calculation using *mm_mpsa.pl* as follows:

(1) $$\begin{array}{l} \Delta {\text{G}}_{\text{MM}/\text{GBSA}}=\Delta {\text{E}}_{\text{vdW}}+\Delta {\text{E}}_{\text{ele}}+\Delta {\text{G}}_{\text{pol}}+\Delta {\text{G}}_{\text{nonpol}}\; \end{array}$$

(ΔE_vdW_: van der Waals interactions contribution, ΔE_ele_: electrostatic energy contribution, ΔG_pol_: polar solvent interaction energy calculated by GB model (*igb* = 2), and ΔG_nonpol_: non-polar solvation free energy calculated with LCPO method (0.0072 × ΔΔSASA, SASA was the solvent accessible area with probe radius of 1.4Å).

The per-residue energy contribution between a hSERT residue and ligand was decomposed by:

(2) $$\begin{array}{l} \Delta {\text{G}}_{\text{MM}/\text{GBSA}}^{\text{per}-\text{residue}}=\Delta {\text{E}}_{\text{vdW}}^{\text{per}-\text{residue}}+\Delta {\text{E}}_{\text{ele}}^{\text{per}-\text{residue}}+\Delta {\text{G}}_{\text{pol}}^{\text{per}-\text{residue}}\\ \;+\Delta {\text{G}}_{\text{nonpol}}^{\text{per}-\text{residue}} \end{array}$$

where the first three terms were defined in the same way as the corresponding terms in the Formula 1, and the last term was calculated according to the ICOSA method.

All the analysis of the equilibrium trajectories, including root mean square deviation (RMSD), the representative structures from the dynamic trajectories, binding free energies, were analyzed and predicted via *cpptraj* and *mm_pbsa.pl* programs in AMBER v. 16 (2016). Structural visualization was performed in *PyMOL* software (PyMOL 2.3) (Schrödinger, 2019).

## Results and Discussion

### Chemistry

In this study, the target compounds were designed and synthesized based on the pharmacophores and molecular skeleton of Vorinostat (SAHA). First, the hydroxamic acid group (zinc binding group, *ZBG*) was replaced by novel *ZBGs* (hydroxypropyl amide group and hydroxyethylamino group), and then the surface recognition region (*Cap*) was modified by introducing different substituent groups on the benzene ring, which aimed to discover novel HDAC inhibitors with good inhibitory activities. In addition, all the synthesized compounds were characterized by ^1^H NMR, ^13^C NMR, and ESI-MS (Supplementary Material).

### Analysis of Cytotoxic Activity by MTT

The antitumor activities of the target compounds *in vitro* were firstly evaluated against four different human tumor cell lines including breast lung cancer (A549), cervical cancer (Hela), liver cancer (HepG2), breast cancer (MCF-7), and the normal cell line Human lung fibroblast (WI-38) was selected to assess the cytotoxicity of the synthesized compounds with SAHA as the reference drug. According to the experiment results (Table 1), the synthesized compounds exhibited varied antitumor activities against the selected tumor cell lines, some of which showed considerable inhibitory activity against Hela cells and A549, especially compound **a9** and **b8**. Moreover, it should be noted that all the synthesized compounds showed poor inhibitory activities against the normal cell lines, indicating their lower toxic side effects and relatively high security.

**Table 1:** Anti-proliferative activities of all the synthesized compounds against four tumor cell lines and a normal cell line.

**Compds**.	**R_**1**_**	**R_**2**_**	**IC**_****50****_**(μM)^a^**				
			**A549**	**HepG2**	**Hela**	**MCF-7**	**WI-38**
**a1**	4-F	–CH_3_	42.80 ± 2.41	93.57 ± 4.59	35.99 ± 6.42	60.71 ± 4.56	141.98 ± 4.52
**a2**	4-Cl	–CH_3_	45.8 ± 1.68	88.7 ± 2.34	35.3 ± 3.22	58.9 ± 1.02	119.64 ± 3.43
**a3**	2-Cl	–CH_3_	43.8 ± 7.82	73.6 ± 4.34	30.4 ± 2.38	64.3 ± 4.21	162.14 ± 1.89
**a4**	4-Br	–CH_3_	51.04 ± 2.23	65.54 ± 3.69	31.39 ± 0.94	56.71 ± 10.01	198.89 ± 4.23
**a5**	4-I	–CH_3_	45.6 ± 2.81	68.9 ± 1.95	35.8 ± 2.43	63.2 ± 4.32	157.66 ± 3.16
**a6**	4-CH_3_	–CH_3_	39.2 ± 3.88	74.9 ± 2.05	**19.6** **±** **3.92**	44.8 ± 2.01	97.24 ± 6.36
**a7**	4-NO_2_	–CH_3_	43.8 ± 4.82	88.3 ± 8.64	25.8 ± 8.93	58.7 ± 3.86	210.81 ± 4.32
**a8**	4-N(CH_3_)_2_	–CH_3_	38.6 ± 0.92	65.4 ± 4.32	29.6 ± 3.22	34.5 ± 1.68	200.05 ± 2.83
**a9**	4-CF_3_	–CH_3_	25.86 ± 1.46	33.36 ± 0.95	**11.15** **±** **3.24**	49.87 ± 7.77	95.16 ± 3.16
**a10**	4-C_6_H_5_	–CH_3_	**19.8** **±** **2.11**	26.8 ± 3.22	38.4 ± 1.01	48.4 ± 2.21	125.9 ± 7.83
**a11**	naphthalene	–CH_3_	26.6 ± 2.81	29.2 ± 3.22	21.8 ± 2.42	38.4 ± 2.12	108.7 ± 4.63
**a12**	[d] [1,3] dioxole	–CH_3_	28.4 ± 2.01	37.6 ± 2.89	35.6 ± 0.89	44.6 ± 4.62	146.96 ± 3.82
**b1**	H	–H	33.11 ± 0.96	84.50 ± 1.33	90.37 ± 2.21	94.30 ± 15.32	194.30 ± 5.32
**b2**	4-F	–H	49.53 ± 0.98	65.51 ± 0.84	60.44 ± 1.76	61.47 ± 8.64	140.07 ± 7.05
**b3**	4-Cl	–H	49.26 ± 6.39	82.72 ± 3.09	95.17 ± 1.79	52.70 ± 13.94	152.70 ± 3.94
**b4**	4-Br	–H	44.02 ± 2.93	66.71 ± 3.34	55.89 ± 4.06	65.05 ± 15.02	191.69 ± 5.16
**b5**	4-I	–H	48.33 ± 0.92	90.31 ± 8.18	60.98 ± 5.31	64.47 ± 11.05	130.05 ± 5.02
**b6**	4-CH_3_	–H	26.94 ± 4.79	77.22 ± 4.43	59.71 ± 2.67	99.19 ± 1.90	144.47 ± 8.05
**b7**	3,5-dimethyl	–H	32.32 ± 3.13	87.98 ± 9.39	96.77 ± 2.09	83.61 ± 23.16	183.61 ± 7.16
**b8**	4-C_6_H_5_	–H	26.50 ± 0.26	28.43 ± 0.65	**13.68** **±** **1.31**	40.07 ± 7.05	95.93 ± 4.28
**b9**	4-CF_3_	–H	**18.97** **±** **1.49**	22.08 ± 0.53	23.02 ± 1.24	91.69 ± 5.16	98.67 ± 7.67
**b10**	[d] [1,3] dioxole	–H	28.23 ± 0.87	33.90 ± 2.71	38.09 ± 1.28	93.25 ± 3.53	193.25 ± 6.53
**b11**	4-N(CH_3_)_2_	–H	32.85 ± 0.78	36.29 ± 1.21	46.78 ± 2.26	35.93 ± 4.28	99.19 ± 1.90
**b12**	naphthalene	–H	33.42 ± 2.12	37.55 ± 6.14	76.38 ± 6.78	98.67 ± 7.67	161.47 ± 8.64
**SAHA**			4.85 ± 0.22	4.95 ± 0.13	4.75 ± 0.15	6.09 ± 1.32	9.09 ± 1.32

a *Data are shown as mean ± SD of three experiments. The compounds with promising inhibitory activities are highlighted in bold*.

The target compounds with bulky substituents in the *Cap* generally showed good inhibitory activities toward Hela and A549, such as compound **a9**, **a10**, **a11**, **b8**, and **b9**, indicating the inhibitory activities of the synthesized compounds was sensitive to the *Cap* modification and the larger Cap groups could better interact with the amino acids at the entrance of the binding pocket than those with smaller Cap groups.

### Analysis of Apoptosis and Cycle Arrest of Hela Cells Induced by Compounds a9 and b8

**Figure 2:**
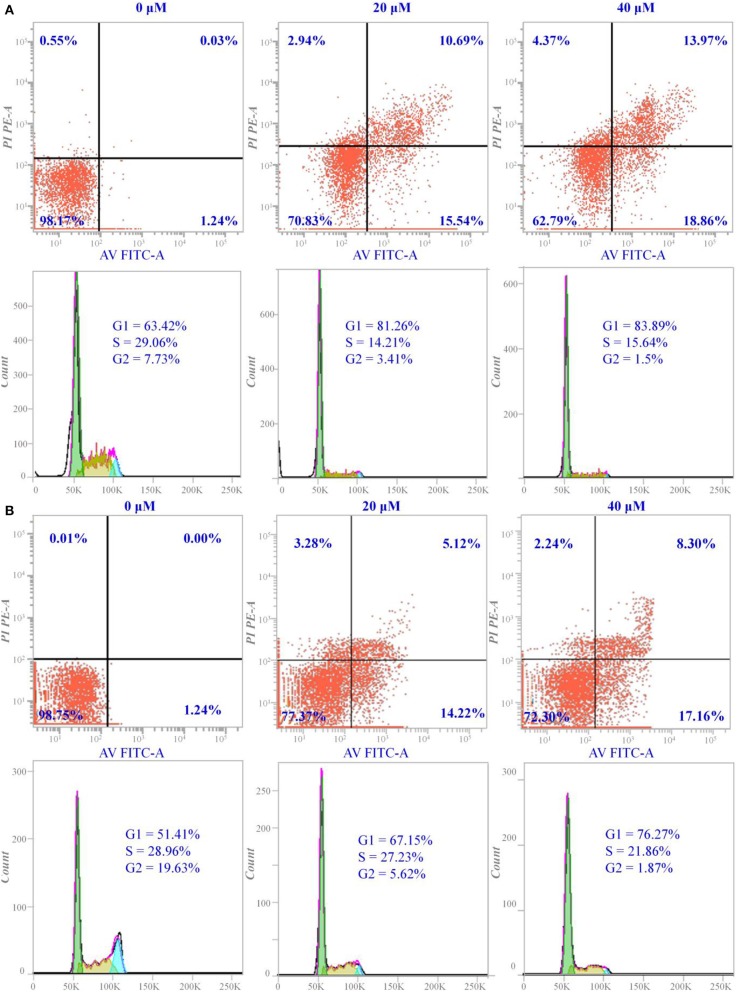
Apoptosis and cell cycle arrest of Hela cells induced by compounds **a9** and **b8**: **(A)** the cells treated with different concentrations of compound **a9** for 48 h; **(B)** the cells treated with different concentrations of compound **b8** for 48 h.

For the majority of anticancer drugs, antitumor pharmacological mechanism was associated with inducing apoptosis, and many HDAC inhibitors have been reported to inhibit proliferation by interfering cell cycle. Thus, it is necessary to explore whether the cytotoxic effects of the synthesized compounds were induced through blocking the cell cycle during mitosis. According to Figure 2, it could be learnt that compound **a9** and **b8** could induce early- and late- apoptosis. For compound **a9**, the proportion of early apoptotic Hela cells was 15.54 and 18.86%, and the proportion of late apoptotic cells was 10.69 and 13.97%, when the Hela cells treated with compound **a9** at concentrations of 20 and 40 μM, respectively, indicating that inhibitory activities of the compound were positively correlated with its concentration. In addition, the inhibitory activities of compound **b8** on Hela cells were also positively correlated with its concentration. When the concentration of compound **b8** increased to 40 μM, the number of early-apoptotic and late-apoptotic cells increased accordingly. However, unlike compound **a9**, the number of late apoptosis induced by compound **a8** was relatively small, and compound **a9** had stronger pro-apoptotic effect than that of compound **b8**, which was consistent with the experimental results of MTT assay.

In addition, cycle block of the compounds **a9** and **b8** with different concentrations on Hela cells was assessed by flow cytometer, and the experimental results showed that the two compounds could block the cell proliferation in the G1 phase. The number of Hela cells stagnated in G1 ranged from 67.15 to 76.27% after the treatment with various concentrations of compound **b8**, suggesting that compound **b8** blocked the cell cycle in G1 phase by the dose-dependent manner (Figure 2). Moreover, the number of Hela cells stagnated in G1 was not sensitive to the concentration of the compound **a9**, and the number of Hela cells blocked in G1 phase accounted for 81.26% when treated by compound **a9** with the low concentration (20 μM). Thus, compounds **a9** and **b8** could exhibit antitumor activities by blocking the G1 phase of the cell cycle, and compounds **a9** showed good anti-tumor activities due to the more Hela cells stagnated in G1.

### Enzyme Inhibition Assay

Hela nuclear extracts was rich in Class I HDACs (especially HDAC1) and contained low concentrations of Class IIa, Class IIb, and Class IV isoforms, which were applied to assess the inhibitory effects of compounds **a9** and **b8** on the enzyme targets. In this study, due to the similar pharmacophore and molecular skeleton to SAHA, SAHA was selected as the reference drug. Although the bioactivities of compounds **a9** and **b8** on the Hela nuclear extracts were lower than that of SAHA, both of them were at the level of micromole range and also had promising inhibitory effects (Table 2). Particularly, compound **a9** showed certain inhibitory activities (IC_50_ = 5.5 ± 2.8 μM), which was the main reason for its strong cytotoxicity to Hela cells.

**Table 2:** Inhibitory activities against the HDACs extracted from Hela cervical cancer cells.

**Compounds**	**Hela nuclear extract IC_**50**_ (μM)^a^**
SAHA	0.367 ± 0.012
**a9**	5.5 ± 2.8
**b8**	22.5 ± 5.2

a *Data are shown as mean ± SD of three experiments*.

Moreover, the inhibitory bioactivities against HDAC1 and HDAC6 subtypes were also determined, and according to Table 3, it could be found that compound **a9** had promising inhibitory activities against HDAC1&6 (HDAC1: IC_50_ = 5.30 ± 1.31 μM; HDAC6: IC_50_ = 8.90 ± 1.90 μM), suggesting its certain broad-spectrum inhibitory activities on HDAC family. However, the inhibitory activity of compound **b8** on HDAC6 was more than ten folds that of HDAC1, indicating its selective inhibitory activities against HDAC6 (HDAC1: IC_50_ = 56.5 ± 2.70 μM; HDAC6: IC_50_ = 4.20 ± 1.27 μM).

**Table 3:** HDAC Enzyme Activity Data for Compound **a9** and **b8**.

**Comp**	**IC_**50**_^a^ (μM)**	
	**HDAC1**	**HDAC6**
SAHA	0.333 ± 0.01	0.475 ± 0.012
**a9**	5.30 ± 1.31	8.90 ± 1.90
**b8**	56.5 ± 2.70	4.20 ± 1.27

a *IC_50_ values were obtained based on three independent experiments and expressed as mean ± SD*.

### Western Blot Analysis

**Figure 3:**
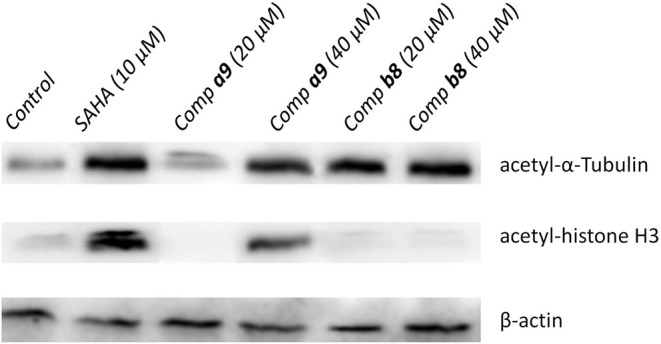
Effects of compounds **a9** and **b8** on acetylation of histone H3 and α-tubulin with SAHA (5 μM) as the reference drug.

Due to the certain inhibitory activities of the compounds **a9** and **b8** against HDACs, it was important to explore the expression level of the substrates of HDACs, which was necessary to elucidate the inhibitory mechanism of the tested compounds. According to Figure 3, the acetylation level of histone H3 could be up-regulated by SAHA and compound **a9**, which was insensitive to the treatment of compound **b8**. In addition, SAHA, compound **a9**, and compound **b8** can increase the acetylation level of α-Tubulin. Based on the knowledge of HDAC families, it could be learnt that histone H3 was the main catalytic substrate of HDAC Class I family and α-Tubulin was the catalytic substrate of HDAC6 (HDAC Class II), indicating the broad-spectrum inhibitory activities of compound **a9** and the selective inhibition of compound **b8** against HDAC6, which was consistent with the results of enzyme inhibition assay.

### Constructing the Studied Systems via Molecular Docking

**Figure 4:**
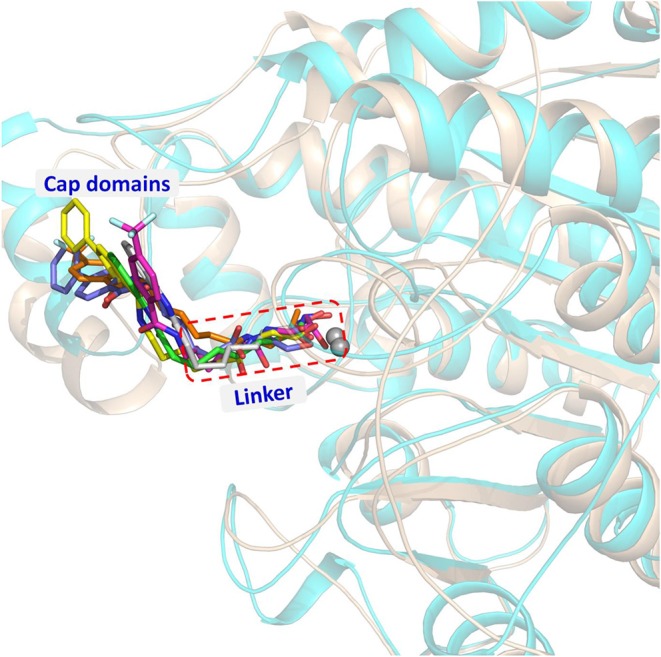
Structural alignment of the initial docking poses of the studied systems: (1) SAHA-HDAC1 (green color); (2) SAHA-HDAC6 (light gray); (3) compound **a9-**HDAC1 (magentas); (4) compound **a9-**HDAC6 (orange); (5) compound **b8-**HDAC1 (magentas); (4) compound **b8-**HDAC6 (light blue).

On the basis of the protein crystals of HDAC1&6 in PDB, molecular docking was applied to build the initial conformations of HDAC1&6 with compound **a9** and **b8**, and the spatial similarity to the ligands inherent in the original crystals and the docking score were the main criterion for selecting the starting conformations. In addition, in this study, the difference in the binding patterns of the synthesized compounds in HDAC1&6 were studied. As shown in Figure 4, it could be found that the *Linker* groups and the ZBGs of the ligands were highly consistent in the orientation at the binding pocket of HDAC1&6, while there were some differences in the spatial orientation of the *Cap* groups of the docked ligands. Overall, the conformations of the docked ligands were very similar in the active pocket.

### MD Simulation

#### Assessment of the Simulation Stability via RMSD Analysis

**Figure 5:**
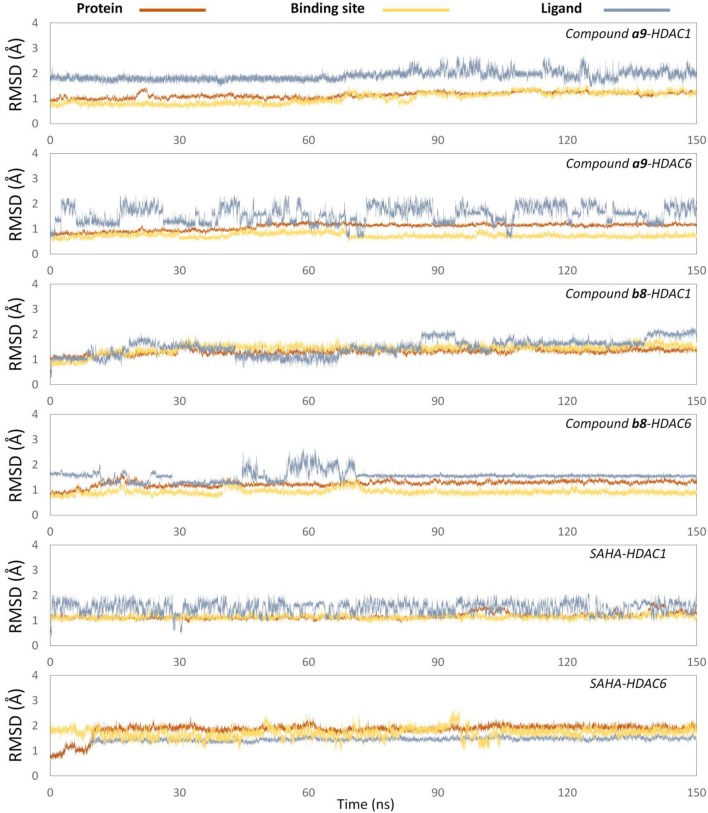
Root mean square deviations of protein backbone atoms, ligand heavy atoms, and binding site residue backbone atoms as a function of time in MD simulations.

**Figure 6:**
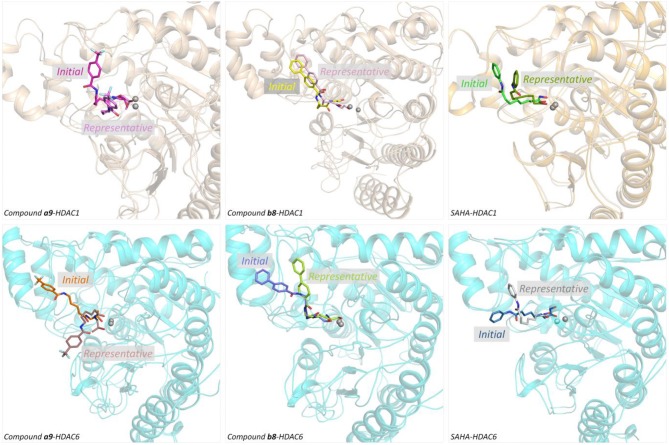
Structural superimposition of the initial docking poses and the representative conformations of all the studied systems.

In order to further explore the interactions between the receptor and ligands, the initial conformations of the constructed studied systems obtained from molecular docking were first subjected to 150 ns MD simulation, and the RMSD values of the backbone atoms of protein, and the heavy atoms of residues consisting of the binding pocket, and the heavy atoms of ligands were used to monitor the dynamic stabilities of the studied systems. All the systems could reach equilibrium state around 100 ns with slight fluctuations, and the docked ligands underwent a certain deflection (≈ 2.0 Å) during molecular dynamics simulation to interact with amino acids in the pocket (Figure 5). In addition, the spatial shifts of compound **a9** and SAHA were relatively large compared to compound **b8** in HDAC1, and the ligands docked to HDAC6 underwent a relatively large deflection during the MD simulation process in order to make the ligands bind to the receptor more stably (Figure 6).

#### Identifying the Key Residues for the Target-Ligand Interactions

**Figure 7:**
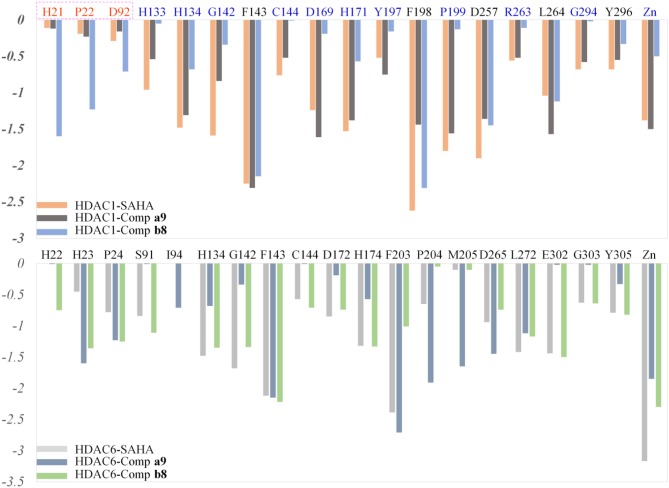
Identification of the key residues in HDAC1&6 with energy contributions to the docked ligands' binding by per-residue binding free energy decomposition.

HDACs belonged metal-protein families, and the zinc ion played vital role in maintaining the stability of catalytic center locating in the bottom of the active pocket (Zhang et al., 2019). Thus, the interactions between the ligands and zinc ions should also be considered, and in this study, 12-6-4 model was applied to process the parameters of zinc ion. The binding free energies of HDAC1-SAHA, HDAC1-compound **a9**, HDAC-compound **b8**, HDAC6-SAHA, HDAC6-compound **a9**, and HDAC6-compound **b8** calculated by MM/GBSA (Genheden and Ryde, 2015) were −48.93, −40.38, −38.28, 46.37, 38.59, and 42.72 kcal/mol, respectively, which was basically consistent with the experimental activities of MTT assay and enzyme inhibition. In addition, in order to discover the key residues, the decomposition free energy was adopted to quantify the energy contribution of each residues and identify the key ones (|energy contribution| ≥ 0.5 kcal/mol). According to Figure 7, we could find that the per-residue energy contribution of the same amino acid in HDAC1&6 contributed differently to the different ligands (taking H134 in HDAC1 as an example, the energy contributions to SAHA, compound **a9**, and **b8**'s binding were −1.48, −1.31, and −0.68 kcal/mol), and different amino acids contributed differently to the same ligand (taking HDAC1-SAHA systems as an example, the energy contribution of H21 was −2.25 kcal/mol, which was 20 times of H21's contribution). Since SAHA and compound **a9** were highly consistent in spatial conformation, they interacted very similarly with amino acids in the active pocket, such as H133, H134, G142, F143, C144, D169, H171, Y197, P199, etc.

**Figure 8:**
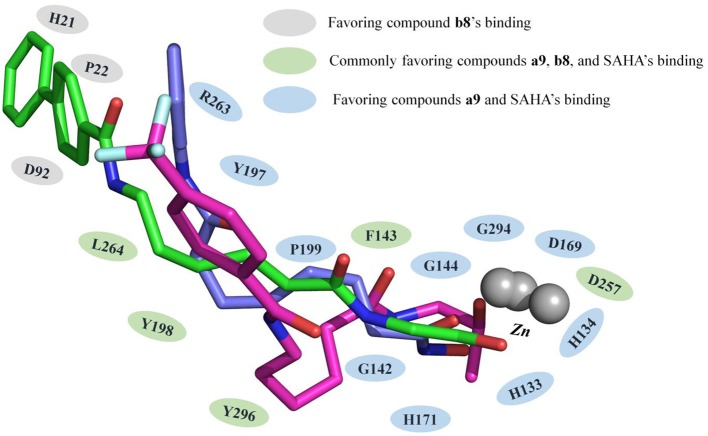
Comparison of compound **a9**, **b8**, and SAHA's binding conformations in HDAC1.

**Figure 9:**
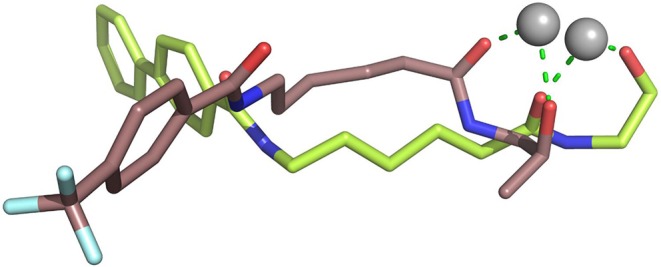
Comparison of the chelation effects between the ZBGs of compound **a9**&**b8** and the zinc ion in HDAC6.

Due to the bulky *Cap* group in compound **b8**, compound **b8** oriented toward Loop1 and Loop2 and interacted strongly with H21, P22, and D92, inducing the conformational changes of *Linker* group in the active pocket. According to Figure 8, it could be found that the number of residues significantly contributing SAHA and compound **a9**'s binding was more than that of compound **b8**, which led to the decrease in the compound **b8**'s binding to HDAC1. In addition, although there were differences in the energy contributions of the amino acids at the docking site of HDAC6 to the docked ligands' binding, the energy complementary between the residues resulted the relatively small difference in the sum of the energy contributions of the residues located in HDAC6 active pocket (SAHA-HDAC6: −21.62 kcal/mol; compound **b8**-HDAC6: −20.49 kcal/mol; compound **a9**-HDAC6: −18.56 kcal/mol), which was consistent with the decreasing gradient of inhibitory activities of the three compounds. Moreover, ZBGs of compound **a9** and compound **b8** formed bi-chelation interactions with zinc ion in the HDAC6 (Figure 9), and the interactions between the zinc ion and ZBGs were −1.85 and 2.30 kcal/mol, indicating their stronger competition with HDAC6 for the zinc ion.

**Figure 10:**
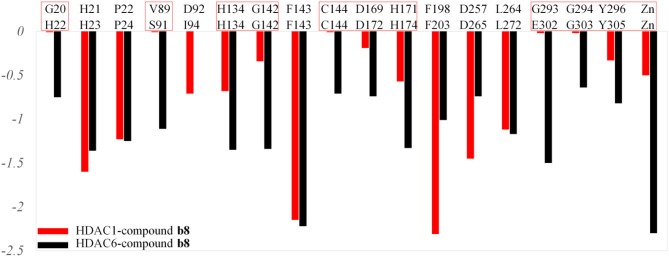
Comparison of the residues with energy contribution to compound **b8**'s binding to HDAC1 and HDAC6.

**Figure 11:**
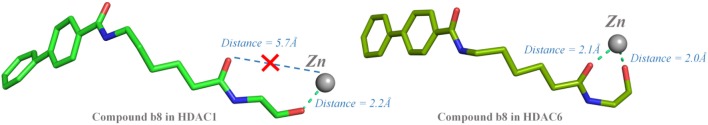
Comparison between the chelating interactions of ZBG in compound **b8** with the zinc ions in HDAC1&6.

Moreover, due to the difference in the effect of compound **b8** on the acetylation levels of histone H3 and α-Tubulin, the binding mechanisms of compound **b8** on HDAC1&6 were further compared. According to Figure 10, conservative and non-conservative amino acids in the active pockets of HDAC1&6 jointly contributed to the difference in compound b8's binding conformations. Among them, the conservative amino acids with large differences in energy contribution were H134/H134, G142/G142, C144/C144, D169/D172, H171/H174, G294/G303, Y296/Y305, and non-conservative amino acids were G20/H22, V89/S91, G293/E302. It should be noted that G20/H22 and V89/S91 were located at the entrance of the active pocket and interact with the *Cap* group of compound **b8**, which would affect the binding conformation of the molecular backbone and then induce the difference in the interaction with receptors. In addition to the differences in interactions with the residues in the active pockets of HDAC1&6, the interactions between the ZBG and the zinc ion were also different. In the HDAC1-compound **b8** system, ZBG formed a single chelation interaction with zinc ion, while formed bi-chelation interactions in HDAC6 system (Figure 11). Compared to the single chelation, the bi-chelation interactions could better compete with the proteins for the zinc ion, which was consistent with the results of enzyme inhibition assay and western blot analysis.

## Conclusion

In this study, 24 HDAC inhibitors with novel ZBG were designed, synthesized, and evaluated for their bioactivities, and computational approaches were applied to investigate the molecular mechanism underlying the compounds' enzyme inhibition. All the tittle compounds were characterized by ^1^H NMR, ^13^C NMR, and ESI-MS. In addition, compound **a9** and compound **b8** had a certain inhibitory activity on tumor cell lines, especially Hela cells, and the target compounds exhibited quite weak toxicity to the normal cell line. The enzyme inhibition experiment and Western blot test showed that compound a9 had certain broad-spectrum inhibitory activities on the HDAC family, while compound **b8** exhibited strong selective inhibition against HDAC6, providing insights for the design of new HDAC inhibitors.

## Data Availability Statement

All datasets generated for this study are included in the article/Supplementary Material.

## Author Contributions

DH and YZ conceived the work and directed the experiments. JH, SW, XL, RL, FD, ZJ, and CZ performed the molecular docking and MD simulation. ZL, HZ, LT, QJ, and PY analyzed the experimental results. DH and YZ drafted the first and second version of the manuscript. All authors read, edited, and approved the final version of manuscript.

### Conflict of Interest

The authors declare that the research was conducted in the absence of any commercial or financial relationships that could be construed as a potential conflict of interest.
