# Supplementary material for: Synthesis and Biological Evaluation of HDAC Inhibitors With a Novel Zinc Binding Group
Source: Front Chem. 2020 Apr 15;8:256. doi: 10.3389/fchem.2020.00256 (PMC7174758; doi:10.3389/fchem.2020.00256)
Supplement: Supplementary file 1 [file Data_Sheet_1.docx]

**Synthesis and Biological Evaluation of HDAC Inhibitors with a Novel Zinc Binding Group**

**Junquan He^1, 2^, Songsong Wang^5^, Xingang Liu^6^, Ruili Lin^1, 2^, Fang Deng^1^, Zhong Jia^4^, Chenghong Zhang^1^, Zhao Li^1^, Hongtian Zhu^1^, Lei Tang^1^, Pingrong Yang^1, 2^, Dian He^1, 4^*, Qingzhong Jia^6*^, Yang Zhang^3, 6*^**

^1^ Materia Medica Development Group, Institute of Medicinal Chemistry, Lanzhou University School of Pharmacy, Lanzhou 730000, China

^2^ NMPA Key Laboratory for Quality Control of Traditional Chinese Medicine, Gansu Institute for Drug Control, Lanzhou 730070, China

^3^ School of Pharmaceutical Sciences, Chongqing University, Chongqing 401331, China

^4^ Pharmacy Department, Lanzhou Second People's Hospital, Lanzhou 730000, China

^5^ The Second Hospital of Hebei Medical University, Shijiazhuang, 050000, China.

^6^ College of Pharmacy, Hebei Medical University, Shijiazhuang 050017, China

* Corresponding authors:

Hed@lzu.edu.cn (D. He);

zhangy2016@cqu.edu.cn (Y. Zhang);

**Spectrum Information**

**4-fluoro-N-(6-((2-hydroxypropyl) amino)-6-oxohexyl) benzamide (a1)**

White solid, yield of 45.6%; m.p. 99.3-100.8℃; ^1^H NMR (400 MHz, DMSO-*d*_6_) δ 8.45 (t, *J* = 5.1 Hz, 1H), 7.89 (dd, *J* = 8.8, 5.6 Hz, 2H), 7.72 (t, *J* = 5.2 Hz, 1H), 7.28 (t, *J* = 8.9 Hz, 2H), 4.63 (s, 1H), 4.10 (s, 1H), 3.60 (d, *J* = 5.7 Hz, 1H), 3.25 – 3.19 (m, 3H), 2.08 (t, *J* = 7.4 Hz, 2H), 1.56 – 1.47 (m, 4H), 1.30 – 1.23 (m, 2H), 0.98 (d, *J* = 6.2 Hz, 3H). ^13^C NMR (101 MHz, DMSO) δ 173.21, 172.68, 165.47, 131.60, 130.18 (d, J_C-F_ = 9.0 Hz), 115.56 (d, J_C-F_ = 22 Hz), 65.75, 49.06, 46.79, 35.77, 29.35, 26.64, 25.69, 21.54. ESI-MS: calcd for C_16_H_23_FN_2_O_3_ [M+H]^+^ 311.1803, found 311.1803. [2M+H]^+^ 621.3509, found 621.3509.

**4-chloro-N-(6-((2-hydroxypropyl) amino)-6-oxohexyl) benzamide (a2)**

White solid, yield of 47.8%; m.p. 93.9-95.7℃; ^1^H NMR (400 MHz, DMSO-*d*_6_) δ 8.51 (s, 1H), 7.85 (d, *J* = 8.3 Hz, 2H), 7.72 (s, 1H), 7.53 (d, *J* = 8.3 Hz, 2H), 4.62 (d, *J* = 4.2 Hz, 1H), 4.10 (s, 1H), 3.60 (s, 1H), 3.23 (d, *J* = 6.4 Hz, 3H), 2.08 (t, *J* = 7.3 Hz, 2H), 1.51 (p, *J* = 7.2 Hz, 4H), 1.30 – 1.24 (m, 2H), 0.98 (d, *J* = 6.0 Hz, 3H). ^13^C NMR (101 MHz, DMSO) δ 172.64, 165.44, 136.25, 133.87, 129.52, 128.76, 65.74, 49.06, 46.79, 35.76, 29.31, 26.64, 25.55, 21.55. ESI-MS: calcd for C_16_H_23_ClN_2_O_3_ [M+H]^+^ 327.1510, found 327.1510. [2M+Na]^+^ 675.2753, found 675.2753.

**2-chloro-N-(6-((2-hydroxypropyl) amino)-6-oxohexyl) benzamide (a3)**

White solid, yield of 53.6%; m.p. 110.2-113.6℃; ^1^H NMR (400 MHz, Methanol-*d*_4_) δ 7.51 – 7.37 (m, 4H), 3.87 – 3.78 (m, 1H), 3.38 (t, *J* = 6.9 Hz, 2H), 3.23 (dd,*J* = 13.5, 4.9 Hz, 1H), 3.12 (dd, *J* = 13.5, 6.9 Hz, 1H), 2.26 (t, *J* = 7.5 Hz, 2H), 1.67 (ddd, *J* = 14.7, 12.4, 7.3 Hz, 4H), 1.50 – 1.42 (m, 2H), 1.15 (dd, *J* = 6.5, 3.6 Hz, 3H). ^13^C NMR (101 MHz, MeOD) δ 175.00, 168.64, 136.49, 130.65, 130.46, 129.56, 128.42, 126.72, 66.00, 46.34, 39.23, 35.51, 28.59, 25.27, 24.87, 19.60. ESI-MS: calcd for C_16_H_23_ClN_2_O_3_ [M+H]^+^ 327.0324, found 327.0324. [2M+Na]^+^ 675.1060, found 675.1060.

**4-bromo-N-(6-((2-hydroxypropyl) amino)-6-oxohexyl) benzamide (a4)**

Tan solid, yield of 43.5%; m.p. 112.4-114.7℃; ^1^H NMR (400 MHz, DMSO-*d*_6_) δ 8.45 (t, *J* = 5.1 Hz, 1H), 7.89 (dd, *J* = 8.8, 5.6 Hz, 2H), 7.72 (t, *J* = 5.2 Hz, 1H), 7.28 (t, *J* = 8.9 Hz, 2H), 4.63 (s, 1H), 4.10 (s, 1H), 3.60 (d, *J* = 5.7 Hz, 1H), 3.25 – 3.19 (m, 3H), 2.08 (t, *J* = 7.4 Hz, 2H), 1.56 – 1.47 (m, 4H), 1.30 – 1.23 (m, 2H), 0.98 (d, *J* = 6.2 Hz, 3H). ^13^C NMR (101 MHz, DMSO-*d*_6_) δ 172.65, 165.57, 134.23, 131.69, 131.69, 129.73, 129.73, 125.15, 65.74, 49.06, 35.76, 26.64, 25.69, 25.55, 21.55. ESI-MS: calcd for C_16_H_23_BrN_2_O_3_ [M+H]^+^ 371.1053, found 371.1053. [M+3H]^+^ 373.1053, found 373.1053. [2M+Na]^+^ 765.1834, found 765.1834.

**N-(6-((2-hydroxypropyl) amino)-6-oxohexyl)-4-iodobenzamide (a5)**

White solid, yield of 47.2%; m.p.148.7-150.2℃; ^1^H NMR (400 MHz, DMSO-*d*_6_) δ 8.49 (s, 1H), 7.84 (d, *J* = 8.4 Hz, 2H), 7.71 (s, 1H), 7.62 (s, 2H), 5.57 (d, *J* = 8.0 Hz, 1H), 4.62 (s, 1H), 4.22 (s, 1H), 2.96 (t, *J* = 5.8 Hz, 3H), 2.10 – 2.05 (m, 2H), 1.52 – 1.48 (m, 4H), 1.23 (s, 2H), 0.98 (d, *J* = 6.2 Hz, 3H). ^13^C NMR (101 MHz, DMSO) δ 173.22, 166.41, 137.55, 134.62, 129.60, 102.04, 65.74, 46.78, 35.75, 33.81, 29.30, 26.70, 25.69, 21.51. ESI-MS: calcd for C_16_H_23_IN_2_O_3_ [M+H]^+^ 418.9529, found 418.9529.

**N-(6-((2-hydroxypropyl) amino)-6-oxohexyl)-4-methylbenzamide (a6)**

White solid, yield of 50.5%; m.p.114.2-116.8℃; ^1^H NMR (400 MHz, DMSO-*d*_6_) δ 8.34 (s, 1H), 7.73 (d, *J* = 8.1 Hz, 3H), 7.25 (d, *J* = 7.9 Hz, 2H), 4.64 (s, 1H), 4.10 (s, 1H), 3.63 – 3.56 (m, 1H), 3.24 – 3.19 (m, 3H), 2.08 (t, *J* = 7.4 Hz, 2H), 1.56 – 1.45 (m, 4H), 1.32 – 1.23 (m, 2H), 0.98 (d, *J* = 6.2 Hz, 3H). ^13^C NMR (101 MHz, DMSO) δ 172.67, 166.38, 141.21, 132.37, 129.18, 127.58, 65.74, 49.06, 46.79, 35.78, 29.44, 26.67, 25.68, 21.56, 21.39. ESI-MS: calcd for C_17_H_23_N_2_O_3_ [M+H]^+^ 307.2055, found 307.2055.

**N-(6-((2-hydroxypropyl) amino)-6-oxohexyl)-4-nitrobenzamide (a7)**

White solid, yield of 49.2%; m.p.124.3-126.1℃; ^1^H NMR (400 MHz, Methanol-*d*_4_) δ 8.34 (d, *J* = 8.9 Hz, 2H), 8.04 (d, *J* = 8.9 Hz, 2H), 3.86 – 3.76 (m, 1H), 3.42 (t, *J* = 7.1 Hz, 2H), 3.20 (d, *J* = 4.9 Hz, 1H), 3.12 (dd, *J* = 13.5, 6.9 Hz, 1H), 2.26 (t, *J* = 7.5 Hz, 2H), 1.68 (dt, *J* = 14.4, 7.4 Hz, 6H), 1.14 (d, *J* = 6.3 Hz, 4H). ^13^C NMR (101 MHz, MeOD) δ 174.96, 166.64, 149.53, 140.13, 128.24, 123.23, 65.99, 46.32, 39.63, 35.47, 28.68, 25.26, 24.88, 19.59. ESI-MS: calcd for C_16_H_23_N_3_O_5_ [M+H]^+^ 338.0546, found 338.0546.

**4-(dimethylamino)-N-(6-((2-hydroxypropyl) amino)-6-oxohexyl) benzamide (a8)**

White solid, yield of 47.8%; m.p.143.5-144.6℃; ^1^H NMR (400 MHz, DMSO-*d*_6_) δ 10.54 (s, 1H), 8.05 (s, 1H), 7.70 (d, *J* = 8.9 Hz, 2H), 6.68 (d, *J* = 8.9 Hz, 2H), 5.59 – 5.55 (m, 1H), 4.07 (s, 1H), 3.60 (s, 4H), 2.96 (d, *J* = 6.1 Hz, 6H), 2.07 (d, *J* = 7.4 Hz, 2H), 1.52 (s, 4H), 1.23 (s, 2H), 0.99 (d, *J* = 5.1 Hz, 3H). ^13^C NMR (101 MHz, MeOD) δ 172.53, 167.64, 154.53, 130.13, 130.14, 111.23, 111.23, 68.32, 49.06, 41.32, 41.32, 39.63, 36.47, 29.38, 25.26, 25.88, 25.7, 20.59. ESI-MS: calcd for C_18_H_29_N_3_O_3_ [M+H]^+^ 336.2323, found 336.2323.

**N-(6-((2-hydroxypropyl) amino)-6-oxohexyl)-4-(trifluoromethyl) benzamide (a9)**

White solid, yield of 43.2%; m.p.106.3-108.1℃; ^1^H NMR (400 MHz, Methanol-*d*_4_) δ 8.00 (d, *J* = 8.1 Hz, 2H), 7.80 (d, *J* = 8.2 Hz, 2H), 3.86 – 3.78 (m, 1H), 3.42 (t, *J* = 7.1 Hz, 2H), 3.33 (p, *J* = 1.6 Hz, 2H), 2.26 (t, *J* = 7.5 Hz, 2H), 1.68 (d, *J* = 7.3 Hz, 4H), 1.43 (t, *J* = 7.7 Hz, 2H), 1.14 (d, *J* = 6.3 Hz, 3H). ^13^C NMR (101 MHz, MeOD) δ 175.00, 173.62, 167.31, 138.14, 127.64, 125.15, 125.11, 125.07, 122.57, 65.99, 46.31, 39.54, 35.48, 28.71, 25.27, 24.87, 19.57. ESI-MS: calcd for C_17_H_23_F_3_N_2_O_3_ [M+H]^+^ 361.1781, found 361.1781. [2M+H]^+^ 721.3453, found 721.3453.

**N-(6-((2-hydroxypropyl) amino)-6-oxohexyl)-[1, 1’-biphenyl]-4-carboxamide (a10)**

White solid, yield of 46.9%; m.p.140.3-142.6℃; ^1^H NMR (400 MHz, DMSO-d6) δ 10.53 (s, 1H), 8.48 (s, 1H), 7.93 (d, J = 8.4 Hz, 2H), 7.74 (dd, J = 12.5, 7.8 Hz, 5H), 7.49 (t, J = 7.5 Hz, 2H), 7.39 (d, J = 7.4 Hz, 1H), 6.05 (s, 1H), 4.22 (t, J = 6.6 Hz, 1H), 3.59 (s, 1H), 2.97 (t, J = 5.9 Hz, 3H), 2.09 (t, J = 7.3 Hz, 2H), 1.57 – 1.48 (m, 4H), 1.33 – 1.26 (m, 2H), 0.99 (d, J = 6.2 Hz, 3H). ^13^C NMR (101 MHz, DMSO) δ 172.68, 166.17, 142.99, 139.69, 133.96, 129.49, 128.46, 128.27, 127.31, 126.91, 65.75, 49.06, 46.79, 35.79, 29.41, 26.67, 25.69, 21.56. ESI-MS: calcd for C_22_H_28_N_2_O_3_ [M+H]^+^ 369.2218, found 369.2218.

**N-(6-((2-hydroxypropyl) amino)-6-oxohexyl)-2-naphthamide (a11)**

White solid, yield of 41.9%; m.p.111.6-113.6℃; ^1^H NMR (400 MHz, DMSO-*d*_6_) δ 8.60 (s, 1H), 8.43 (s, 1H), 8.05 – 7.88 (m, 4H), 7.73 (s, 1H), 7.64 – 7.55 (m, 2H), 5.57 (d, *J* = 8.0 Hz, 1H), 4.63 (s, 1H), 4.10 (s, 1H), 3.60 (s, 1H), 2.97 (t, *J* = 5.9 Hz, 2H), 2.10 (t, *J* = 7.4 Hz, 2H), 1.53 (dd, *J* = 15.8, 7.9 Hz, 4H), 1.34 – 1.26 (m, 2H), 0.98 (d, *J* = 6.3 Hz, 3H). ^13^C NMR (101 MHz, DMSO) δ 172.65, 165.59, 149.92, 147.70, 129.18, 122.47, 108.22, 107.69, 102.04, 65.75, 49.06, 46.79, 35.78, 29.41, 26.66, 25.70, 21.56. ESI-MS: calcd for C_20_H_26_N_2_O_3_ [M+H]^+^ 343.2063, found 343.2063.

**N-(6-((2-hydroxypropyl) amino)-6-oxohexyl) benzo [d][1,3] dioxole-5-carboxamide (a12)**

White solid, yield of 42.0%; m.p.123.6-125.3℃; ^1^H NMR (400 MHz, DMSO-*d*_6_) δ 10.54 (s, 1H), 8.26 (s, 1H), 7.72 (s, 1H), 7.48 – 7.32 (m, 2H), 6.97 (d, *J* = 8.1 Hz, 1H), 6.08 (s, 2H), 4.62 (d, *J* = 4.6 Hz, 1H), 4.04 (s, 0H), 3.61 (dd, *J* = 11.0, 5.9 Hz, 1H), 3.19 (dt, *J* = 11.5, 5.8 Hz, 3H), 2.08 (t, *J* = 7.4 Hz, 2H), 1.56 – 1.44 (m, 4H), 1.26 (dt, *J* = 14.8, 7.4 Hz, 2H), 0.99 (d, *J* = 6.2 Hz, 3H). ^13^C NMR (101 MHz, DMSO) δ 172.65, 165.59, 149.92, 147.70, 129.18, 122.47, 108.22, 107.69, 102.04, 65.75, 49.06, 46.79, 35.78, 29.41, 26.66, 25.70, 21.56. ESI-MS: calcd for C_17_H_24_N_2_O_5_ [M+H]^+^ 337.1800, found 337.1800.

**N-(6-((2-hydroxyethyl) amino)-6-oxohexyl) benzamide (b1)**

Off-white solid, yield of 41%; m.p.111.2-114.5℃; ^1^H NMR(400 MHz, DMSO-*d_6_*, TMS, ppm): *δ* 8.40 (t, 1H, *J*= 6.0Hz, -ArCONH-), 7.80-7.77(m, 2H, Ar-H_2_, Ar-H_6_), 7.73(t, 1H, *J*=4.0Hz, -CONH-), 7.49-7.39(m, 3H, Ar-H_3_, Ar-H_4_, Ar-H_5_), 4.60(t, 1H, *J*=4.0Hz, -OH), 3.35-3.30(dd, 2H, *J_1_*=8.0Hz, *J_2_*=12.0Hz, -CH_2_OH), 3.22-3.17(dd, 2H, *J_1_*=8.0Hz, *J_2_*=12.0Hz, -CH_2_NH-), 3.07-3.03(dd, 2H, *J_1_*=4.0Hz, *J_2_*=12.0Hz, -CH_2_NH-), 2.03(t, 2H, *J*= 6.0Hz, -CH_2_CO- ), 1.50-1.43(m, 4H, -CH_2_-, -CH_2_-), 1.27-1.19(m, 2H, -CH_2_-). ^13^C-NMR (100 MHz, DMSO-*d_6_*, TMS, ppm):172.66, 166.53, 135.19, 131.43, 128.68, 127.58, 60.44, 41.86, 39.77, 35.78, 29.41, 26.67, 25.52. ESI-MS: calcd for C_15_H_22_N_2_O_3_ [M+H]^+^ 279.1630, found 279.1744.

**2 4-fluoro-N-(6-((2-hydroxyethyl) amino)-6-oxohexyl) benzamide (b2)**

Faint yellow solid, yield of 51%; m.p.131.2-132.9℃; ^1^H NMR(400 MHz, DMSO-*d_6_*, TMS, ppm): *δ* 8.43 (t, 1H, *J*= 5.6Hz, -ArCONH-), 7.88-7.83(m, 2H, Ar-H_2_, Ar-H_6_), 7.73(t, 1H, *J*=6.0Hz, -CONH-), 7.28-7.22(m, 2H, Ar-H_3_, Ar-H_5_), 4.60(t, 1H, *J*=5.6Hz, -OH), 3.35-3.30(dd, 2H, *J_1_*=6.0Hz, *J_2_*=12.0Hz, -CH_2_OH), 3.20-3.16(dd, 2H, *J_1_*=6.8Hz, *J_2_*=12.8Hz, -CH_2_NH-), 3.07-3.02(dd, 2H, *J_1_*=6.0Hz, *J_2_*=12.0Hz, -CH_2_NH-), 2.02(t, 2H, *J*= 7.6Hz, -CH_2_CO-), 1.50-1.43(m, 4H, -CH_2_-, -CH_2_-), 1.26-1.19(m, 2H, -CH_2_-).  ^13^C-NMR (100 MHz, DMSO-*d_6_*, TMS, ppm):172.64, 165.46, 162.97, 130.23, 130.14, 115.68, 60.44, 41.85, 35.77, 29.37, 26.65, 25.50. ESI-MS: calcd for C_15_H_21_FN_2_O_3_ [M+H]^+^ 297.1536, found 297.1622.

**4-chloro-N-(6-((2-hydroxyethyl) amino)-6-oxohexyl) benzamide (b3)**

Faint yellow solid, yield of 43%; m.p.112.4-115.9℃; ^1^H NMR(400 MHz, DMSO-*d_6_*, TMS, ppm): *δ* 8.52 (t, 1H, *J*= 5.6Hz, -ArCONH-), 7.85(d, 2H, *J*= 8.0Hz, Ar-H_2_, Ar-H_6_), 7.77(t, 1H, *J*=5.2Hz, -CONH-), 7.53(d, 2H, *J*= 8.0Hz, Ar-H_3_, Ar-H_5_), 4.64(s, 1H, -OH), 3.38-3.33(dd, 2H, *J_1_*=8.0Hz, *J_2_*=12.0Hz, -CH_2_OH), 3.25-3.20(dd, 2H, *J_1_*=7.2Hz, *J_2_*=12.0Hz, -CH_2_NH-), 3.11-3.06(dd, 2H, *J_1_* = 8.0Hz, *J_2_*=12.0Hz, -CH_2_NH-), 2.06(t, 2H, *J*= 8.0Hz, -CH_2_CO-), 1.54-1.47(m, 4H, -CH_2_-, -CH_2_-), 1.30-1.24(m, 2H, -CH_2_-). ^13^C-NMR (100 MHz, DMSO-*d_6_*, TMS, ppm):172.65, 165.46, 136.27, 133.88, 129.53, 128.77, 60.44, 41.85, 39.63, 35.76, 29.32, 26.65, 25.69. ESI-MS: calcd for C_15_H_21_N_2_O_3_ [M+H]^+^ 313.1241, found 313.1317.

**4-bromo-N-(6-((2-hydroxyethyl) amino)-6-oxohexyl) benzamide (b4)**

White solid, yield of 35% ; m.p.115.7-118.7℃; ^1^H NMR(400 MHz, DMSO-*d_6_*, TMS, ppm): *δ* 8.49 (t, 1H, *J*= 5.6Hz, -ArCONH-), 7.74(t, 1H, *J*=5.4Hz, -CONH-), 7.73-7.72(m, 2H, Ar-H_2_, Ar-H_6_), 7.64-7.62(m, 2H, Ar-H_3_, Ar-H_5_), 4.62-4.59(m, 1H, -OH), 3.35-3.21(dd, 2H, *J_1_*=5.6Hz, *J_2_*=12.8Hz, -CH_2_OH), 3.19-3.14(dd, 2H, *J_1_*=6.4Hz, *J_2_*=12.4Hz, -CH_2_NH-), 3.01-2.97(dd, 2H, *J_1_*=5.6Hz, *J_2_*=11.2Hz, -CH_2_NH-), 2.02(t, 2H, *J*= 7.2Hz, -CH_2_CO-), 1.49-1.42(m, 4H, -CH_2_-, -CH_2_-), 1.24-1.19(m, 2H, -CH_2_-). ^13^C-NMR (100 MHz, DMSO-*d_6_*, TMS, ppm):172.62, 165.41, 133.25, 131.27, 129.67, 126.85, 60.43, 40.61, 39.72, 36.45, 29.61, 25.66, 25.21. ESI-MS: calcd for C_15_H_21_BrN_2_O_3_ [M+H]^+^ 357.0736, found 357.0806 and 359.0825.

**N-(6-((2-hydroxyethyl) amino)-6-oxohexyl)-4-iodobenzamide (b5)**

White solid, yield of 33%; m.p.150.4-150.9℃; ^1^H NMR(400 MHz, DMSO-*d_6_*, TMS, ppm): *δ* 8.47 (t, 1H, *J*= 5.6Hz, -ArCONH-), 7.80(d, 2H, *J*= 8.4Hz, Ar-H_2_, Ar-H_6_), 7.73(t, 1H, *J*=5.2Hz, -CONH-), 7.57(d, 2H, *J*= 8.8Hz, Ar-H_3_, Ar-H_5_), 4.62-4.59(m, 1H, -OH), 3.34-3.21(dd, 2H, *J_1_*=6.0Hz, *J_2_*=12.0Hz, -CH_2_OH), 3.20-3.16(dd, 2H, *J_1_*=7.2Hz, *J_2_*=12.8Hz, -CH_2_NH-), 3.07-3.02(dd, 2H, *J_1_*=6.0Hz, *J_2_*=12.0Hz, -CH_2_NH-), 2.02(t, 2H, *J*= 7.2Hz, -CH_2_CO-), 1.49-1.42(m, 4H, -CH_2_-, -CH_2_-), 1.24-1.18(m, 2H, -CH_2_-). ^13^C-NMR (100 MHz, DMSO-*d_6_*, TMS, ppm):172.61, 166.67, 137.57, 133.21, 129.19, 97.75, 60.41, 41.65, 39.46, 36.23, 29.20, 25.89, 25.30. ESI-MS: calcd for C_15_H_21_IN_2_O_3_ [M+H]^+^ 405.0597, found 405.0692.

**N-(6-((2-hydroxyethyl) amino)-6-oxohexyl)-4-methylbenzamide (b6)**

White solid, yield of 45%; m.116.5-118.5℃; ^1^H NMR(400 MHz, DMSO-*d_6_*, TMS, ppm): *δ* 8.31 (t, 1H, *J*= 5.6Hz, -ArCONH-), 7.72(t, 1H, *J*=5.2Hz, -CONH-), 7.69(d, 2H, *J*= 8.0Hz, Ar-H_2_, Ar-H_6_), 7.21(d, 2H, *J*= 8.0Hz, Ar-H_3_, Ar-H_5_), 4.59(s, 1H, -OH), 3.34-3.21(dd, 2H, *J_1_*=6.0Hz, *J_2_*=12.4Hz, -CH_2_OH), 3.20-3.15(dd, 2H, *J_1_*=7.2Hz, *J_2_*=13.0Hz, -CH_2_NH-), 3.07-3.03(dd, 2H, *J_1_*=6.0Hz, *J_2_*=11.8Hz, -CH_2_NH-), 2.30(s, 3H, -CH3), 2.02(t, 2H, *J*= 7.0Hz, -CH_2_CO-), 1.50-1.43(m, 4H, -CH_2_-, -CH_2_-), 1.26-1.19(m, 2H, -CH_2_-). ^13^C-NMR (100 MHz, DMSO-*d_6_*, TMS, ppm):172.52, 165.65, 134.31, 131.75, 129.79, 125.22, 61.29, 51.79, 35.27, 29.36, 26.70, 25.55, 21.43. ESI-MS: calcd for C_16_H_24_N_2_O_3_ [M+H]^+^ 293.1787, found 293.1868.

**N-(6-((2-hydroxyethyl) amino)-6-oxohexyl)-3,5-dimethylbenzamide (b7)**

White solid, yield of 52%; m.p.110.1-111.8℃; ^1^H NMR(400 MHz, DMSO-*d_6_*, TMS, ppm): *δ* 8.27 (t, 1H, *J*= 5.6Hz, -ArCONH-), 7.72(t, 1H, *J*= 5.6Hz, -CONH-), 7.39(s, 2H, Ar-H_2_, Ar-H_6_), 7.10(s, 1H, Ar-H_4_), 4.59(s, 1H, -OH), 3.34-3.29(dd, 2H, *J_1_*=6.4Hz, *J_2_*=12.0Hz, -CH_2_OH), 3.20-3.14(dd, 2H, *J_1_*=7.0Hz, *J_2_*=13.0Hz, -CH_2_NH-), 3.07-3.02(dd, 2H, *J_1_*=5.8Hz, *J_2_*=12.2Hz, -CH_2_NH-), 2.26(s, 6H, -CH_3_,-CH_3_), 2.02(t, 2H, *J*= 7.6Hz, -CH_2_CO-), 1.48-1.42(m, 4H, -CH_2_-, -CH_2_-), 1.25-1.19(m, 2H, -CH_2_-). ^13^C-NMR (100 MHz, DMSO-*d_6_*, TMS, ppm):172.65, 166.33, 138.54, 137.47, 134.32, 128.14, 61.23, 41.93, 39.55, 36.17, 29.54, 25.85, 25.43, 21.02. ESI-MS: calcd for C_17_H_26_N_2_O_3_ [M+H]^+^ 307.1943, found 307.2024.

**N-(6-((2-hydroxyethyl) amino)-6-oxohexyl)-[1, 1'-biphenyl]-4-carboxamide (b8)**

White solid, yield of 36%; m.p.147.6-148.5℃; ^1^H NMR(400 MHz, DMSO-*d_6_*, TMS, ppm): *δ* 8.46 (t, 1H, *J*= 5.6Hz, -ArCONH-), 7.89-7.87(m, 2H, Ar_1_-H_2_, Ar_1_-H_6_), 7.73-7.71(m, 3H, -CONH-, Ar_1_-H_3_, Ar_1_-H_5_), 7.70-7.67 (m, 2H, Ar_2_-H_2_, Ar_2_-H_6_), 7.47-7.43(m, 2H, Ar_2_-H_3_, Ar_2_-H_5_), 7.38-7.36(m, 1H, Ar_2_-H_4_), 4.60(t, 1H, *J*= 5.2Hz, -OH), 3.35-3.31(dd, 2H, *J_1_*=5.6Hz, *J_2_*=12.0Hz, -CH_2_OH), 3.19-3.14(dd, 2H, *J_1_*=7.2Hz, *J_2_*=12.0Hz, -CH_2_NH-), 3.01-2.96(dd, 2H, *J_1_*=5.6Hz, *J_2_*=11.6Hz, -CH_2_NH-), 2.03(t, 2H, *J*= 7.6Hz, -CH_2_CO-), 1.51-1.42(m, 4H, -CH_2_-, -CH_2_-), 1.26-1.15(m, 2H, -CH_2_-). ^13^C-NMR (100 MHz, DMSO-*d_6_*, TMS, ppm):172.65, 166.16, 144.03, 139.71, 133.98, 129.48, 128.46, 128.28, 127.32, 126.92, 60.45, 41.86, 35.79, 29.43, 25.42, 24.93. ESI-MS: calcd for C_21_H_26_N_2_O_3_ [M+H]^+^ 355.1943, found 355.2025.

**N-(6-((2-hydroxyethyl) amino)-6-oxohexyl)-4-(trifluoromethyl) benzamide (b9)**

White solid, yield of 45%; m.p.105.0-106.3℃; ^1^H NMR(400 MHz, DMSO-*d_6_*, TMS, ppm): *δ* 8.65 (t, 1H, *J*= 5.6Hz, -ArCONH-), 7.99(d, 2H, *J*= 8.0Hz, Ar-H_2_, Ar-H_6_), 7.81(d, 2H, *J*= 8.0Hz, Ar-H_3_, Ar-H_5_), 7.73(t, 1H, *J*=5.8Hz, -CONH-), 4.61(s, 1H, -OH), 3.34-3.29(dd, 2H, *J_1_*=6.4Hz, *J_2_*=12.8Hz, -CH_2_OH), 3.24-3.19(dd, 2H, *J_1_*=7.2Hz, *J_2_*=12.8Hz, -CH_2_NH-), 3.07-3.02(dd, 2H, *J_1_*=6.0Hz, *J_2_*=11.6Hz, -CH_2_NH-), 2.03(t, 2H, *J*= 7.2Hz, -CH_2_CO-), 1.52-1.43(m, 4H, -CH_2_-, -CH_2_-), 1.27-1.19(m, 2H, -CH_2_-). ^13^C-NMR (100 MHz, DMSO-*d_6_*, TMS, ppm):172.63, 165.37, 138.92, 131.57, 128.51, 125.76, 125.73, 60.44, 41.85, 39.8, 35.75, 29.25, 26.63. 25.48. ESI-MS: calcd for C_16_H_21_F_3_N_2_O_3_ [M+H]^+^ 347.1504, found 347.1583.

**N-(6-((2-hydroxyethyl) amino)-6-oxohexyl) benzo[d][1,3] dioxole-5-carboxamide (b10)**

White solid, yield of 37%; m.p.94.5-96.7℃; ^1^H NMR(400 MHz, DMSO-*d_6_*, TMS, ppm): *δ* 8.24 (t, 1H, *J*= 5.6Hz, -ArCONH-), 7.73(t, 1H, *J*=5.6Hz, -CONH-), 7.39-7.37(m, 1H, Ar-H_6_), 7.33 (d, 1H, *J*=2.0Hz, Ar-H_2_), 6.93(d, 1H, *J*=8.4Hz, Ar-H_5_), 6.04(s, 2H, -OCH_2_O-), 4.61-4.59(m, 1H, -OH), 3.35-3.30(dd, 2H, *J_1_*=6.0Hz, *J_2_*=12.0Hz, -CH_2_OH), 3.18-3.15(dd, 2H, *J_1_*=6.8Hz, *J_2_*=12.8Hz, -CH_2_NH-), 3.07-3.02(dd, 2H, *J_1_*=6.0Hz, *J_2_*=12.0Hz, -CH_2_NH-), 2.02(t, 2H, *J*= 7.6Hz, -CH_2_CO-), 1.50-1.41(m, 4H, -CH_2_-, -CH_2_-), 1.25-1.17(m, 2H, -CH_2_-). ^13^C-NMR (100 MHz, DMSO-*d_6_*, TMS, ppm):172.65, 166.63, 152.32, 148.19, 128.46, 120.82, 114.73, 111.92, 101.42, 60.34, 41.55, 39.54, 36.35, 29.38, 25.48, 25.16. ESI-MS: calcd for C_16_H_22_N_2_O_5_ [M+H]^+^ 322.1529, found 322.1546.

**4-(dimethylamino)-N-(6-((2-hydroxyethyl) amino)-6-oxohexyl) benzamide (b11)**

Faint yellow solid, yield of 42%; m.p.120.4-122.3℃; ^1^H NMR(400 MHz, DMSO-*d_6_*, TMS, ppm): *δ* 8.01 (t, 1H, *J*= 5.6Hz, -ArCONH-), 7.70(t, 1H, *J*=5.6Hz, -CONH-), 7.67-7.65(m, 2H , Ar-H_2_, Ar-H_6_), 6.66-6.63(m, 2H, Ar-H_3_, Ar-H_5_), 4.58(t, 1H, *J*=5.4Hz, -OH), 3.35-3.31(dd, 2H, *J_1_*=6.0Hz, *J_2_*=12.0Hz, -CH_2_OH), 3.18-3.13(dd, 2H, *J_1_*=6.8Hz, *J_2_*=12.8Hz, -CH_2_NH-), 3.07-3.03(dd, 2H, *J_1_*=6.0Hz, *J_2_*=12.0Hz, -CH_2_NH-), 2.91(s, 6H, -N(CH_3_)_2_), 2.02(t, 2H, *J*= 7.2Hz, -CH_2_CO-), 1.50-1.40(m, 4H, -CH_2_-, -CH_2_-), 1.25-1.18(m, 2H, -CH_2_-). ^13^C-NMR (100 MHz, DMSO-*d_6_*, TMS, ppm):172.68, 167.70, 154.51, 130.23, 123.71, 111.29, 60.29, 41.56, 41.43, 41.73, 39.78, 36.45, 29.61, 25.56, 25.41. ESI-MS: calcd for C_17_H_27_N_3_O_3_ [M+H]^+^ 322.2052, found 322.2136.

**N-(6-((2-hydroxyethyl) amino)-6-oxohexyl)-2-naphthamide (b12)**

White solid, yield of 50%; m.p.115.1-117.8℃; ^1^H NMR(400 MHz, DMSO-*d_6_*, TMS, ppm): *δ* 8.45 (t, 1H, *J*= 5.4Hz, -ArCONH-), 8.38(s, 1H, Ar-H_1_), 7.98-7.87(m, 4H, Ar-H_3_, Ar-H_4_, Ar-H_5_, Ar-H_8_), 7.59(t, 1H, *J*= 5.6Hz, -CONH-), 7.58-7.54 (m, 2H, Ar-H_6_, Ar-H_7_), 4.47(t, 1H, *J*= 5.6Hz, -OH), 3.37-3.33(dd, 2H, *J_1_*=6.0Hz, *J_2_*=12.0Hz, -CH_2_OH), 3.30-3.25(dd, 2H, *J_1_*=7.2Hz, *J_2_*=9.2Hz, -CH_2_NH-), 3.09-3.05(dd, 2H, *J_1_*=6.0Hz, *J_2_*=12.0Hz, -CH_2_NH-), 2.05(t, 2H, *J*= 7.0Hz, -CH_2_CO-), 1.55-1.47(m, 4H, -CH_2_-, -CH_2_-), 1.33-1.26(m, 2H, -CH_2_-). ^13^C-NMR (100 MHz, DMSO-*d_6_*, TMS, ppm):172.63, 166.46, 135.62, 134.12, 132.45, 129.55, 128.36, 128.85, 128.43, 127.27, 126.89, 124.42, 61.62, 41.43, 39.31, 36.84, 29.21, 25.49, 25.06. ESI-MS: calcd for C_19_H_24_N_2_O_3_ [M+H]^+^ 329.1787, found 329.1828.
